# The Activation of Endogenous Proteases in Shrimp Muscle Under Water-Free Live Transport

**DOI:** 10.3390/foods13213472

**Published:** 2024-10-30

**Authors:** Jia Li, Yuxin Liu, Huanhuan Yang, Luyun Cai, Wenqian Nong, Weiliang Guan

**Affiliations:** 1 Department of Food Science, Guangxi University, Nanning 530004, China; 2316392007@st.gxu.edu.cn (J.L.); 2316392010@st.gxu.edu.cn (Y.L.); 2316392020@st.gxu.edu.cn (H.Y.); 2 Ningbo Innovation Center, College of Biosystems Engineering and Food Science, Zhejiang University, Ningbo 315100, China; cailuyun@zju.edu.cn; 3 Institute of Oncology, Guangxi Academy of Medical Sciences, Nanning 530021, China

**Keywords:** *Penaeus vannamei*, endogenous proteases, water-free transport, flesh quality

## Abstract

Water-free transportation (WFT) causes shrimp (*Penaeus vannamei*) flesh quality deterioration. However, the roles of endogenous protease-induced protein hydrolysis have been neglected in the research. In the present study, calpain zymography, gelatinase zymography, the hematoxylin–eosin staining method, and other methods were applied to investigate the response of various endogenous proteases (cathepsin, calpain, and gelatinase), the myofibril fragmentation index (MFI), and the microscopic morphology of shrimp muscle during WFT in comparison with the shrimp under the conventional water transportation strategy (WT). The results showed that the total activity of proteases in shrimp muscle increased significantly (*p* ≤ 0.05) after simulated transportation. Cathepsins and gelatinases were activated during WFT. No significant (*p* > 0.05) changes of the activity of caspase-3 and the muscle cell apoptosis rate were detected in shrimp muscle cells after WFT. In addition, the MFI increased and the gap among muscle fiber bundles enlarged after WFT. Compared with WFT, no significant (*p* > 0.05) effect on the activities of calpain, gelatinase, and caspase-3 in the muscle of shrimp was found after WT, and only the activity of cathepsin L significantly increased (*p* ≤ 0.05). Based on the findings, we concluded that the activation of various endogenous proteases was induced during WFT.

## 1. Introduction

The Pacific white shrimp (*Penaeus vannamei*) is widely favored due to its superior taste and nutritional properties, and it is also the most economically valuable cultured shrimp species in the world [1]. The amount of *Penaeus vannamei* cultured in China in 2022 was 1,340,280 tons, with an increment of 5.23% compared with 1,273,632 tons in 2021 [2].

Commonly cited criteria for shrimp quality degradation include a loss of gloss and spots, a loss of chewiness and a loss of nutrients [3]. Although refrigeration storage helps maintain the quality of shrimp, there is a strong preference for live shrimp in the Chinese market. This preference arises from the significant quality degradation that occurs due to protein autolysis and polyphenol oxidation within just a few hours postmortem. The flesh quality and nutritional value of live shrimp far exceed those of frozen and chilled products [4,5]. The water-free transportation (WFT) strategy was recently developed for transporting live shrimp from farm to market [6,7]. WFT has the characteristics of low weight and high transportation efficiency. However, shrimp suffer various stresses under WFT, especially air exposure and low temperature stress [8]. The antemortem stress of animals has been proven to be involved in flesh quality. In our previous studies, we observed that the oxidation of lipids and proteins, along with metabolic disorders, which led to a deterioration in flesh quality, manifested as increased drip loss and texture softening after WFT [4,9]. In addition to oxidation and metabolism, endogenous protease-induced flesh autolysis also plays a great role in flesh quality deterioration; endogenous proteases expose hydrophobic amino acids in a low pH environment, resulting in increased surface hydrophobicity, which affects drip loss raising [10]. The activity of these proteases is associated with physiological responses to stress [11]. The activation of endogenous proteases leads to a decrease in shrimp meat quality, which leads to a decrease in purchases by consumers. Therefore, it is necessary to study the activation of endogenous proteases during WFT.

It is generally believed that cathepsins, calpains, and gelatinases in muscle tissue are endogenous proteases, which are sensitive to environmental stressors in the muscles of aquatic animals such as fish and shrimp [12,13,14]. Cathepsins (cathepsin B and cathepsin L) and calpains (μ-calpain and m-calpain) mainly act on the myofibrillar protein (MP). The cathepsins in animal muscle belong to acid protease, which exist in the lysosome of muscle cells. The increase in intracellular calcium and the acidic substances produced by anaerobic metabolisms, such as lactate and phosphoric acid, causes the decrease in pH, and thereafter activates the phospholipase, which leads to the decomposition of phospholipids in the lysosomal membrane [15,16]. As a result, the permeability of the lysosome membrane increases, and lysosomal enzymes (including cathepsins) are released, leading to the proteolysis of muscle tissue [17]. The calpains are sensitive to increments of intracellular Ca^2+^ [18]. In addition, gelatinases are involved in a family of matrix metalloproteinases (MMPs) and mainly act on the intramuscular connective tissue connecting myofibrils, which are activated by Ca^2+^; their activities also increase with the decrease in muscle pH [9]. A previous study found that fast pH decline promoted apoptosis and then caspase-3 activation during postmortem aging [19]. In addition, a low temperature, hypoxia, and other stressors will lead to an increase in the apoptosis rate of muscle tissue [20,21]. Caspase-3 activates and modulates the release of calpain and cathepsin L enzymes and catalyzes the degradation of intracellular proteins of muscle tissue [22].

Guan et al. [4] found that air exposure stress during WFT induced the dysfunction of the Na^+^/Ca^2+^ exchanger and the Ca^2+^ channel of myocyte, leading to intracellular Ca^2+^ overload. In addition, the muscle glycogen in shrimp is converted into lactic acid through anaerobic glycolysis and accumulates in the muscle during WFT. Simultaneously, ATP is degraded, and a large number of ions are produced during this process, which ultimately leads to a decrease in the pH value of the muscle [9].

Based on these findings, we speculate that endogenous proteases might be activated due to the disturbed physiological environment, and are potentially associated with the flesh quality deterioration of shrimp under air exposure stress during WFT. In this study, changes in the endogenous protease (cathepsins, calpains, gelatinases, and caspase-3) activity in shrimp muscle during WFT were focused on; meanwhile, the traditional live transport strategy (i.e., water transportation) was also employed for reference. The results provide some novel insights into the mechanisms of flesh quality declines of shrimp under transport stress other than protein oxidation and substance metabolism.

## 2. Materials and Methods

### 2.1. Chemicals

Acrylamide, ammonium persulfate, Ac-DEVD-pNA, bovine hemoglobin, bromophenol blue, CaCl_2_, casein, citric acid, dithiothreitol, Ethylene Diamine Tetraacetie Acid (EDTA), gelatin, glycerol, glycine, mercaptoethanol, NaCl, NaH_2_PO_4_, Na_2_HPO_4_, KCl, N,N′-methylene-bisacrylamide (BIS), N,N,N’,N’-Tetramethylethylenediamine (TEMED), persulfuric acid, paraformaldehyde, tri-chloroacetic acid (TCA), Triton X-100, sodium dodecyl sulfate (SDS), xylene, 7-amido-4-methylcoumarin (AMC) and α-thioglycerol were purchased from Shanghai Aladdin Biochemical Technology Co., Ltd. (Shanghai, China). Z-arginine-arginine-7-amido-4-methylcoumarin (Z-Arg-Arg-MCA) and Z-phenylalanine-arginine-7-amino-4-methylcoumarin(Z-PHE-ARG-MCA) were purchased from Sigma Aldrich (St. Louis, MO, USA).

### 2.2. Shrimp and Experiment Design

Live shrimp (*Penaeus vannamei*, weight: 15 ± 2 g, length: 14 ± 1 cm) were obtained from a local market in Hangzhou, China. The shrimp were temporarily cultured for acclimatization in 40 L of seawater (25 °C, 35‰ salinity, 5 mg/L dissolved oxygen) with a culture density of 65 g/L for 24 h.

The shrimp were randomly divided into three groups, control, water-free transportation (WFT), and water transportation (WT), after temporary acclimatization. In the control group, the live shrimp were cultured at 25 °C (without mimicked transportation treatment) at a density of 60 g/L for 10 h. In the WFT group, 2 kg of shrimp (n ≈ 130) were cold-shocked at 13 °C for 3 min, then packaged in a 4 L polyethylene (PE) bag filled with 95% (*v*/*v*) oxygen and stored at 15 °C for 10 h [23]. In the WT group, live shrimp were treated according to the most common commercial live transportation strategy. Two kilograms of shrimp were placed in a water tank containing 8 L of seawater (loading density of 250 g/L), continuously aerated at 25 °C for 10 h. All experiments were performed in triplicate.

### 2.3. Sample Collection

Samples were taken after the simulated transportation and then slaughtered by beheading. Shrimp muscle was collected by peeling the shrimp, and the first segment was cut off as the flesh sample. The collected muscle samples were immediately frozen in liquid nitrogen (−80 °C, 1 min) and stored at −80 °C before testing.

### 2.4. Determination of Total Protease Activity in Muscle

The total activity of protease in shrimp muscle was detected using the method described by Zhang et al. [23]. One gram of muscle sample was homogenized with 4 mL of pre-cooled distilled water in an ice bath, then centrifuged at 16,000× *g* and 4 °C for 30 min, and the supernatant was collected as total protease. A total of 0.4 mL of supernatant was added into 1.2 mL of phosphate–citric acid buffered solution (0.67 mL 0.2 M Na_2_HPO_4_, 0.53 mL 0.1 M citric acid, pH = 5.5), then mixed with 0.4 mL of 1% (*w*/*v*) bovine hemoglobin. After incubation at 30 °C for 1 h, the reaction was stopped by adding 2 mL of 5% (*w*/*v*) trichloroacetic acid (TCA). The mixture was shaken and cooled at room temperature. The corresponding blanks were prepared identically with each sample, except the TCA which was added to the mixed solution in the end. The test samples and corresponding blanks were centrifuged at 20,000× *g* for 15 min, and the supernatant was collected and measured with a Lowry Protein Assay Kit (Solarbio, Beijing, China). The total protease activity was expressed as the TCA soluble peptides released per gram of muscle sample per hour (mg TCA soluble peptide/g muscle).

### 2.5. Determination of Cathepsin B and L Activity in Muscle

The activity of cathepsin B in muscle was detected using a method previously described by Hultmann et al. [24]. One gram of muscle sample was homogenized with 4 mL of pre-cooled distilled water with an ice bath, then centrifuged at 16,000× *g* and 4 °C for 30 min; afterwards, the supernatant was collected as the cathepsin of the muscle samples. The samples were diluted with distilled water to a protein concentration of 2.25 mg/mL. The same volume of buffer (150 mM Bis-Tris, 30 mM Ethylene Diamine Tetraacetie Acid (EDTA), 6 mM dithiothreitol, pH = 7.0) was added to 100 μL of enzyme extract at 4 °C for 10 min, and then 100 μL of substrate (Z-arginine-arginine-7-amido-4-methylcoumarin (Z-Arg-Arg-MCA), 0.1 mM) was added to the mixture. The reaction was stopped by the addition of 3 mL of 1% (*w*/*v*) sodium dodecyl sulfate (SDS) in 50 mM of Bis-Tris (pH = 7.0). The excitation and emission wavelengths were set at 380 nm and 460 nm at 10 nm slits. A standard curve of fluorogenic substance was established by 7-amido-4-methylcoumarin (AMC) as the standard, and the content of AMC in the sample was calculated according to its slope. Activities are given as the amount of AMC released per minute (nmol AMC/min). The detection of cathepsin L activity in the muscle tissue was identical to cathepsin B, except the substrate was Z-phenylalanine-arginine-7-amino-4-methylcoumarin (Z-PHE-ARG-MCA). Cathepsin L activity was calculated by subtracting cathepsin B activity from cathepsin B + L activity.

### 2.6. Determination of Calpain Activity in Muscle

The calpain in shrimp muscle was detected using the method outlined by Wang et al. [25]. The specific operation is as follows.

#### 2.6.1. Extraction of Calpain

Five grams of muscle samples were homogenized with 15 mL of pre-cooled calpain extract buffer (50 mM Tris, 5 mM EDTA, 10 mM α-thioglycerol, pH = 7.4), and the supernatant was collected after centrifuging at 10,000× *g* at 4 °C for 20 min. A volume of 300 μL of the supernatant was mixed with 100 μL of the loading buffer (20% (*v*/*v*) glycerol, 2 mM mercaptoethanol, 0.004% (*w*/*v*) bromophenol blue, 200 mM Tris-HCl, pH = 7.0).

#### 2.6.2. Preparation of Separation Gel Solution and Stacking Gel of Zymogram

Six milliliters of separation gel solution (12% (*w*/*v*) acrylamide, 0.32% (*w*/*v*) N,N′-methylene-bisacrylamide, 375 mM Tris-HCl (pH = 8.8), 0.2% (*w*/*w*) casein, 3.5% glycerol, 0.04% (*w*/*v*) ammonium persulfate, 0.028% (*v*/*v*) TEMED) was poured into the lower layer, and then stored at 37 °C until solidification. Afterwards, the stacking gel solution (4% (*w*/*v*) acrylamide, 0.1% (*w*/*v*) N,N′-methylene-bisacrylamide, 330 mM Tris-HCl (pH = 6.8), 0.08% (*w*/*v*) ammonium persulfate, 0.028% (*v*/*v*) TEMED) was poured over the separation gel, and then stored at 37 °C until solidification.

#### 2.6.3. Electrophoresis

The calpain extractions (10 μL) were loaded into wells for electrophoresis. Electrophoresis was performed at a constant voltage of 125 V using an electrophoresis buffer (25 mM Tris, 192 mM glycine, 1 mM EGTA, 1 mM dithiothreitol, pH = 8.3) with an ice bath for 3 h.

#### 2.6.4. Gel Incubating, Staining, and Analyzing

After electrophoresis, the gel was washed and then incubated overnight in an incubation buffer (4 mM CaCl_2_, 0.1% α-thioglycerol, 50 mM Tris, pH = 7.5) at 20 °C. Subsequently, the gel was washed with a washing buffer (20 mM Tris, 10 mM EDTA, pH = 7.0) for 30 min to remove Ca^2+^. The gel was stained and then destained with a Coomassie Brilliant Blue Gel Staining Kit (Solarbio, Beijing, China) following the manufacturer’s instructions. Afterward, the gel was scanned and analyzed through a gel imaging system (Bio-Rad Laboratories, Hercules, CA, USA). The activity of calpains was indicated by the relative intensity of each band.

### 2.7. Determination of Gelatinase Activity in Muscle

The gelatinases in shrimp muscle were detected using the method outlined by Bao et al. [26]. The specific operation is as follows.

#### 2.7.1. Extraction of Gelatinase

A total of five grams of muscle samples was homogenized with 20 mL of pre-cooled gelatinase extract buffer (1 M Tris, pH = 8.0), then centrifuged at 10,000× *g* at 4 °C for 20 min, and the supernatant was collected. A volume of 300 μL of supernatant was mixed with 100 μL of loading buffer (0.32% (*w*/*v*) Tris-HCl 6.4 mL, 4% (*w*/*v*) SDS (pH = 7.2) 8 mL, 16% (*v*/*v*) glycerol 3.2 mL, bromophenol blue 0.024 g, water 2.4 mL).

#### 2.7.2. Preparation of Separation Gel Solution and Stacking Gel of Zymogram

The separation gel (11.6% (*w*/*v*) acrylamide, 0.4% (*w*/*v*) N,N′-methylene-bisacrylamide, 375 mM Tris-HCl (pH = 8.8), 0.1% (*w*/*v*) SDS, 0.1% (*w*/*v*) persulfuric acid, 0.06% (*v*/*v*) TEMED, 0.1% (*w*/*v*) gelatin) was poured into the lower layer and solidified at 37 °C. Meanwhile, the stacking gel (4.8% (*w*/*v*) acrylamide, 0.2% (*w*/*v*) N,N′-methylene-bisacrylamide, 250 mM Tris-HCl (pH = 6.8), 0.1% (*w*/*v*) SDS, 0.1% (*w*/*v*) persulfuric acid, 0.1% (*v*/*v*) TEMED, 0.1% (*w*/*v*) gelatin) was poured above the separation gel and solidified at 37 °C.

#### 2.7.3. Electrophoresis

Fifteen microliters of gelatinase extract were loaded for electrophoresis with an electrophoresis buffer (25 mM Tris, 0.25 M glycine, 0.1% (*w*/*v*) SDS, pH = 8.3) under a constant current of 30 mA in an ice bath for 4 h.

#### 2.7.4. Gel Incubating, Staining, and Analyzing

After electrophoresis, the gel was washed with a washing buffer (2.5% (*v*/*v*) Triton X-100, 50 mM Tris-HCl, 5 mM CaCl_2_, pH = 7.6) for 30 min to remove SDS, and then incubated in an incubation buffer (50 mM Tris-HCl, 150 mM NaCl, 10 mM CaCl_2_, pH = 7.5) at 37 °C for 24 h. The staining and analyzing methods were according to the description in Section 2.6.4.

### 2.8. Detection of Apoptotic Cells in Muscle

Apoptotic cells in shrimp muscle cells were detected using a TUNEL Apoptosis Detection Kit (Servicebio, Wuhan, China) according to the manufacturer’s instructions. The frozen muscle samples were embedded in a frozen section embedding agent (CM1520, Leica, Wetzlar, Germany) and placed at −80 °C overnight, and then were sliced at −20 °C using a cryostat (CM1520, Leica, Germany) with a slice thickness of 5 μm. The muscle tissue sections were fixed with 4% (*v*/*v*) paraformaldehyde for 30–60 min, then triple-washed in PBS (pH = 7.4). Subsequently, 0.5% (Triton X-100/PBS, *v*/*v*) of Triton X-100 was placed on the section and incubated at room temperature for 5 min, and then washed three times in PBS. The section was covered with one TUNEL staining droplet placed flat in a dark wet box at 37 °C for 2 h. After rinsing three times in PBS, the section was stained by DAPI solution and incubated at room temperature for 10 min in the dark, and then washed three times in PBS. The slides were shaken and washed in PBS (pH = 7.4) 3 times, for 5 min each time. Finally, the section was sealed with an antifade mounting medium and detected using a fluorescence microscope (DMI 3000B, Leica). Imaging was achieved using the excitation and emission wavelengths of DAPI (EX: 350 nm, EM: 420 nm) and TUNEL (EX: 488 nm, EM: 525 nm) dyes.

### 2.9. Determination of Caspase-3 Activity in Muscle

The caspase-3 activity was measured by spectrophotometry using a Caspase-3 Assay Kit for Live Cells (Beyotime Biotechnology, Shanghai, China) according to the manufacturer’s instructions. A total of 100 mg of muscle was added to 1 mL of lysis solution and homogenized with a glass homogenizer in an ice bath. After lysing on ice for 5 min, the lysed muscle tissue was centrifuged at 4 °C and 16,000× *g* for 15 min. The supernatant was mixed with 20 μL of Ac-DEVD-pNA (2 mM) and incubated at 37 °C for 6 h. The optical density (OD) at 405 nm was measured using a microplate reader (Tecan, Mannedorf, Switzerland).

### 2.10. Myofibril Fragmentation Index (MFI)

The myofibril fragmentation index was detected using a method outlined by Zhang et al. [23]. A total of 0.5 g of shrimp meat was homogenized with 30 mL of buffer (0.1 M KCl, 7 mM NaH_2_PO_4_, 18 mM Na_2_HPO_4_, 1 mM EDTA, pH = 7.0) in an ice bath. The homogenate was filtered with two layers of filter paper, and then the filtrate was collected and centrifuged at 10,000× *g* for 10 min at 4 °C. The precipitate was collected and centrifuged with 30 mL of buffer at 10,000× *g* for 10 min at 4 °C. This operation was repeated twice. The final precipitate was resuspended in an appropriate amount of buffer. The protein concentration was adjusted to 0.5 mg/mL using the Biuret method. The MFI was calculated by multiplying the absorbance at 540 nm and 150, an MFI coefficient.

### 2.11. Hematoxylin and Eosin (H and E) Staining

The muscle microstructure of shrimp was observed by hematoxylin–eosin (H and E) staining using a method outlined by Chen et al., with minor modifications [27]. Fresh muscle tissue was fixed in 4% (*v*/*v*) paraformaldehyde for more than 24 h. The tissue was taken out from the fixative and dehydrated in gradient alcohol. The dehydrated samples were transparentized in xylene, and the tissue sections were embedded in paraffin and then serially sectioned into 5 μm-sized portions. The dewaxing sections were stained with H and E and observed through the microscope (DM4000B, Leica, Germany).

### 2.12. Statistical Analysis

The results are expressed as the mean ± standard deviation and all experiments were performed in triplicate. Statistical analyses were carried out using SPSS (version 19.0) using a one-way analysis of variance followed by Duncan’s multiple range test. *p* ≤ 0.05 was considered statistically significant.

## 3. Results

### 3.1. Total Activity of Protease in Muscle

**Figure 1:**
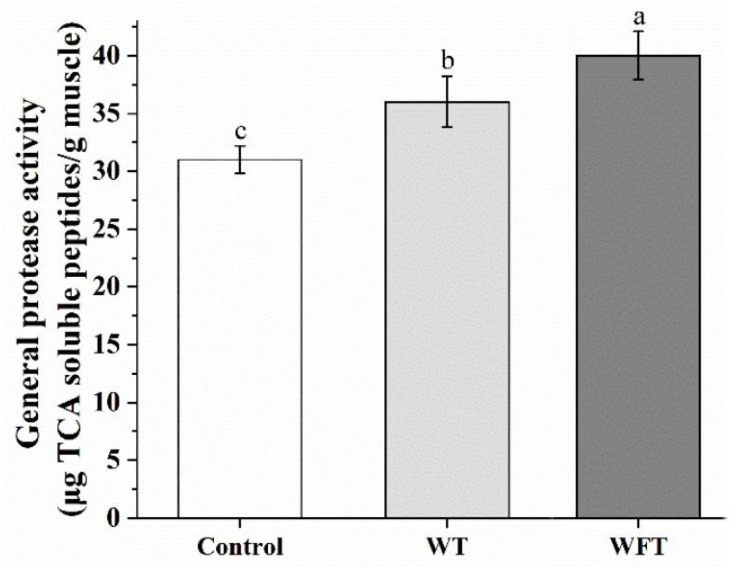
The general protease activity of shrimp muscle from the control group, the mimicked water transportation (WT) group, and the mimicked water-free transportation (WFT) group. Values are presented as the mean ± SD; different letters indicate that significant differences exist (*p* ≤ 0.05).

The concentration of trichloroacetic acid soluble peptide mainly reflects the ability of endogenous proteases in muscle to decompose hemoglobin. As shown in Figure 1, the total activity of endogenous protease in shrimp muscle was significantly increased after simulated transportation. Compared to the WT group, the WFT group exhibited a significantly higher activity of endogenous proteases (*p* ≤ 0.05).

### 3.2. Activity of Cathepsin B and L in Muscle

**Figure 2:**
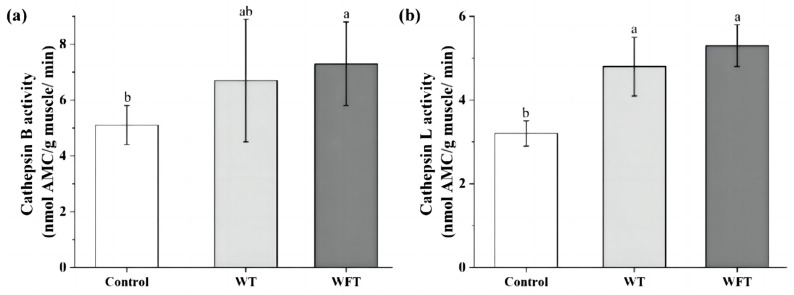
The activity of cathepsin B (**a**) and cathepsin L (**b**) of shrimp muscle from the control group, the mimicked water transportation (WT) group, and the mimicked water-free transportation (WFT) group. Values are presented as the mean ± SD; different letters indicate that significant differences exist (*p* ≤ 0.05).

No significant changes in cathepsin B activity were observed in the WT group compared to the WFT and the control groups (*p* > 0.05, Figure 2a). As shown in Figure 2a,b, both cathepsin B and L activities in WFT showed a significant increase compared to the control group (*p* ≤ 0.05). Conversely, the cathepsin L activity in the WT group showed a significant increase compared to the control group (*p* ≤ 0.05, Figure 2b).

### 3.3. Activity of Calpain in Muscle

**Figure 3:**
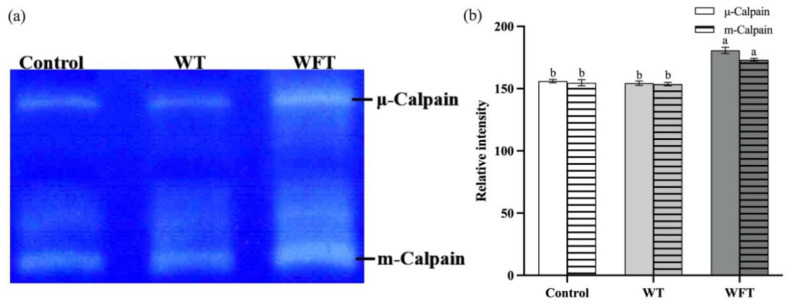
The activity of μ-calpain and m-calpain of shrimp muscle from the control group, the mimicked water transportation (WT) group, and the mimicked water-free transportation (WFT) group. (**a**) Casein zymography; (**b**) the relative intensity of calpain bands; different letters indicate that significant differences of the same calpain exist among treatments (*p* ≤ 0.05).

The calpain in the muscle can decompose the casein in the gel; the white bands in the casein zymogram represent the calpains molecule, and the width of the band is positively correlated with the catalytic activity of the calpain molecule. As exhibited in Figure 3a, μ-calpain and m-calpain were recognized in casein zymography. The activities of μ-calpain and m-calpain in shrimp muscle were both significantly increased after WFT (*p* ≤ 0.05, Figure 3b). In contrast, no significant changes in μ-calpain and m-calpain activities were detected after WT (*p* > 0.05, Figure 3).

### 3.4. Activity of Caspase-3 and Apoptosis in Muscle

**Figure 4:**
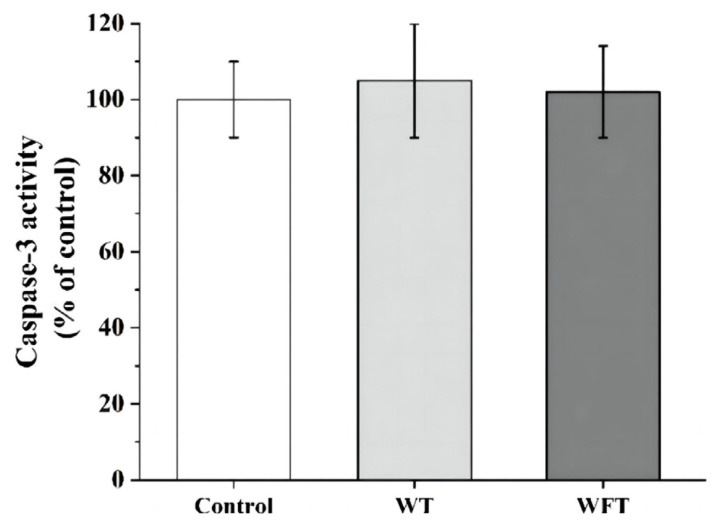
The activity of caspase-3 of shrimp from the control group, the mimicked water transportation (WT) group, and the mimicked water-free transportation (WFT) group. Values are presented as the mean ± SD.

The shrimp exhibited no significant differences in the activity of caspase-3 activity compared to the untreated shrimp after both WFT and WT (control) (*p* > 0.05, Figure 4).

**Figure 5:**
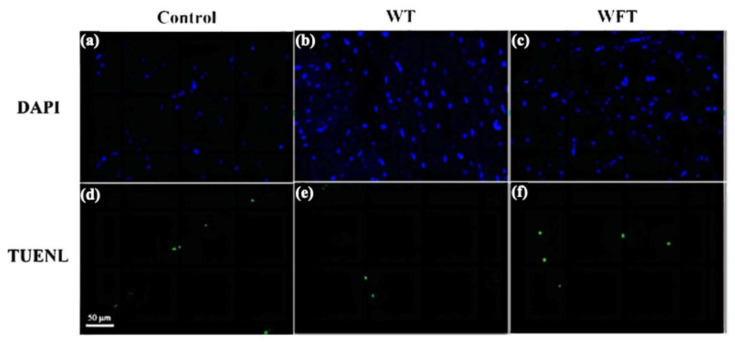
TUNEL assays of muscle tissue of shrimp from the control group, the mimicked water transportation (WT) group, and the mimicked water-free transportation (WFT) group. DAPI (blue) show the nucleus (**a**–**c**), and Fluorescein-dUTp (green) show apoptosis (**d**–**f**).

DAPI labeled all the nuclei, showing blue fluorescence, while TUNEL only labeled apoptotic cells, showing green fluorescence. Similar to the unchanged caspase-3 activity, there was no significant difference in the number of apoptotic cells showing green fluorescence of the control group (Figure 5d), the WT group (Figure 5e), and the WFT group (Figure 5f) of shrimp muscle tissues, demonstrating that the two different simulated transportation strategies did not significantly cause apoptosis of shrimp muscle cells.

### 3.5. Activity of Gelatinase in Muscle

**Figure 6:**
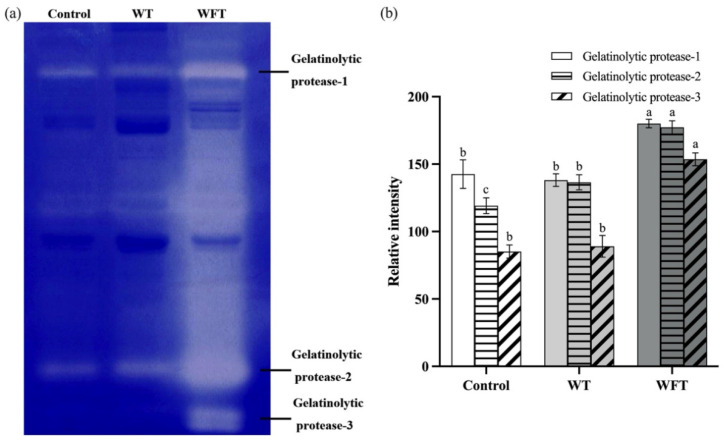
The activity of gelatinolytic proteases of shrimp muscle from the control group, the mimicked water transportation (WT) group, and the mimicked water-free transportation (WFT) group. (**a**) Gelatin zymography; (**b**) the relative intensity of gelatinolytic protease bands; different letters indicate that significant differences of the same gelatinolytic protease exist among treatments (*p* ≤ 0.05).

As shown in the gelatin zymography (Figure 6a), a total of three gelatinolytic proteases (gelatinolytic protease-1, 2, and 3) were identified in the muscle of the WFT group, while only gelatinolytic protease-1 and 2 were observable in the control and WT groups. The activities of all the gelatinolytic proteases in shrimp muscle were remarkably increased after WFT compared to those of the control and WT groups (*p* ≤ 0.05, Figure 6b). In contrast, only the activity of gelatinolytic protease-2 activity was elevated in shrimp muscle after WT (*p* ≤ 0.05, Figure 6b), while the activities of gelatinolytic proteases-1 and 3 remained largely unchanged after WT (*p* > 0.05, Figure 6b).

### 3.6. Myofibril Fragmentation Index and Microstructure of Muscle

**Figure 7:**
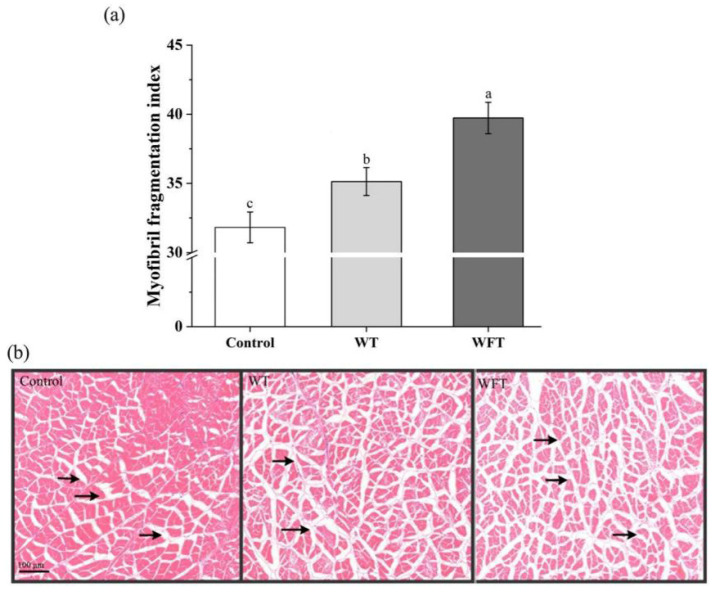
The myofibril fragmentation index (**a**) and cross-sectional micromorphology (**b**) of shrimp muscle. (**a**): The control group, the mimicked water transportation (WT) group, and the mimicked water-free transportation (WFT) group. Values are presented as the mean ± SD; different letters indicate significant differences exist (*p* ≤ 0.05). (**b**) The arrows indicate intermuscular connective tissue.

The myofibril fragmentation index (MFI) of shrimp muscle significantly increased in both WFT and WT compared to the control (*p* ≤ 0.05, Figure 7a), with a more pronounced increase observed in WFT than in WT (*p* ≤ 0.05, Figure 7a).

The cross-sectional micromorphology of the shrimp muscle is shown in Figure 7b. The gap between muscle fibers noticeably widened after both WFT and WT, with a larger gap size in WFT compared to WT. In addition, muscle fiber bundle atrophy was evident following both treatments.

## 4. Discussion

During water-free transportation (WFT), the physiological and biochemical statuses of shrimp undergo a series of changes due to the suffering of diverse stressors, including the increase in intracellular free radical content (reactive oxygen species and reactive nitrogen species), the release of crustacean hyperglycemic hormone (CHH), apoptosis in hemocyte, disturbances in immune and antioxidant systems, and the enhancement of anaerobic metabolism in muscle tissue [4,8,9,28,29]. The overall survival rate after 10 h WFT was above 90%, according to our previous study [4,9]. The oxidation of free radicals and the acidic substances produced by anaerobic metabolism, such as lactate and phosphoric acid, cause damage to the intracellular membrane system, which in turn causes intracellular proteases to be activated or released from lysosomes [16]. The total protease activity refers to the sum of the proteolytic activity of cathepsins, calpains, and gelatinases. In this study, the total activity of proteases in shrimp muscle increased significantly after simulated transportations (Figure 1, *p* ≤ 0.05), indicating that a low temperature and air exposure stress during WFT and crowding and ammonia nitrogen stress during WT could activate endogenous proteases in shrimp muscle. Similar to our results, a previous study found that the total activity of endogenous protease in the muscle of silver carp killed by gill-cutting bloodletting was significantly higher than those killed by head-knockout; although both modes of death were very painful, being killed by gill-cutting bloodletting was more stressful and painful to the silver carp due to its long duration [23].

Cathepsin is an endogenous protease with high hydrolytic activity for myofibrillar protein in muscle. In general, cathepsins are present in the lysosomes of cells, but when animals are under environmental stress, cathepsins are released into the cytoplasm or even extracellularly due to the damaged membrane system [17]. In this work, the activities of cathepsin B and L in shrimp were significantly increased (*p* ≤ 0.05, Figure 2) after WFT, which is probably due to the activities of cathepsins being higher in an acidic environment, and the anaerobic metabolites such as lactate accumulated in the muscle of shrimp, causing a decline in the intracellular pH [30]. These results were in accordance with our previous research, as the pH of shrimp muscle declined after WFT [4]. Similarly, the activities of cathepsin B and L in the muscle of Atlantic salmon (*Sahmo salar* L.) and Atlantic cod (*Gadus morhua*) under low temperatures or crowding stress before slaughter were significantly higher than those without stress [17,24]. The activities of cathepsins were activated in the early stage of storage and gradually increased over time, contributing to the degradation of myofibrillar protein throughout the storage period [23]. This is consistent with the phenomenon of shrimp muscle softening after transportation in our previous study [4].

Both μ-calpain and m-calpain are regarded as the dominant forms within the system and are sensitive to various intracellular biochemical environments, including ionic strength, pH decline, phosphorylation, and oxidation levels [31]. There are few studies on the effect of pre-slaughter stress on calpain activity in the muscle of aquatic animals, but it has been shown that greater stress levels before slaughter were associated with a slower decrease in intact calpain levels and a slower reduction in structural proteins [32]. It is known that the concentration of Ca^2+^ has a significant effect on the activation mechanism of calpains, resulting in the autolysis of myofibril structural proteins and muscle microstructure destruction [33]. In addition, our previous study found that the intracellular Ca^2+^ in shrimp flesh after WFT and WT increased significantly by about four and three times, respectively, compared to the control, and that the intracellular Ca^2+^ level after WFT was significantly higher than after WT [4]. Therefore, we inferred that the elevation of intracellular Ca^2+^ concentration activated calpains during mimicked transportations.

The phenomenon that apoptosis can lead to changes in flesh quality has been discussed in many published articles [34,35]. When apoptosis occurs in muscle cells, the activation of caspase-3 leads to the enzymatic hydrolysis of the cytoskeletal myofibrillar protein, which contributes to muscle softening and drip loss [36]. In the present study, the caspase-3 activity in shrimp muscle was virtually unchanged after WFT and WT (*p* > 0.05, Figure 4), and therefore the apoptosis in muscle cells of shrimp after WFT and WT was also undetected (Figure 5). However, a previous study found that the expression of caspase-3 in *Marsupenaeus japonicus*’s hepatopancreas and gill cells was significantly up-regulated under air exposure at 25 °C after 7.5 h, which was entirely different from our findings [37]. We speculated these results were because the low-temperature treatment can inhibit the apoptosis of shrimp under air exposure stress by reducing the content of ROS in cells, or because the muscles were less sensitive to air exposure stress than the hepatopancreas and gills [8].

Cathepsin, calpain, and caspase-3 in muscle mainly act on the myofibrillar protein, which constitutes muscle fibers, while the substrate of gelatinases is the connective tissue between muscle fibers [17]. Gelatinases in muscle tissue essentially belong to the matrix metalloproteinase family, and their activation pathway is similar to that of calpain, which is also activated by intracellular Ca^2+^ [33]. In addition, the decreased pH condition of muscle is another trigger of the activation of gelatinases [38]. Gelatinase plays a crucial role in the degradation of collagen, leading to antemortem and postmortem softening of the flesh [39]. In this work, the activity of all detected gelatinases in the muscle tissue of shrimp was significantly increased after WFT compared to that of the control and WT groups, while only the activity of gelatinase-2 showed a significant promotion after WT (Figure 6b, *p* ≤ 0.05). As previous studies revealed, the pH level decreased and the Ca^2+^ concentration increased in the shrimp muscle after simulated transportation due to oxidation damage and disrupted metabolism, which were more pronounced in WFT [4]. Consequently, the gelatinases in shrimp muscle were aroused due to the rising of intracellular Ca^2+^ and the declining of pH which resulted from transport stress. Similar to our results, a previous study found that the intracellular Ca^2+^ concentration and the gelatinase activity increase in the muscle of *Anarhichas minor*, under low pH stress [40].

The myofibril fragmentation index (MFI) is an indicator of myofibrillar protein integrity; the myofibrillar protein is an important component of skeletal proteins, and a lower MFI value indicates a lower degree of damage to the integrity of myofibrils, which reflects the level of myofibril breakage [41]. In this study, the MFI of shrimp muscle was significantly increased after WFT compared with the control (Figure 7a, *p* ≤ 0.05). The increase in the degree of myofibril breakage led to the softening of the muscle of the shrimp, which affected the taste and led to a decline in the quality of the shrimp. Other results in this work showed cathepsins and calpains in shrimp muscle were activated during WFT (Figure 2 and Figure 3), and these results indicated that the myofibrillar protein in muscle fibers was hydrolyzed due to the catalysis of cathepsins and calpains [30,42,43]. In addition, this study also found that the gap among muscle fiber bundles in shrimp muscle enlarged after WFT and WT (Figure 7b). The intermuscular connective tissue is composed of collagen, and the activity of gelatinase, which catalyzes the hydrolysis of collagen in shrimp muscle, was significantly increased during WFT (Figure 6). Therefore, the intermuscular connective tissue is hydrolyzed under the action of gelatin protease, leading to the weakening of the binding force between muscle fibers, and ultimately resulting in the gap enlargement among muscle fiber bundles and the atrophy of muscle fiber bundles [44]. The phenomenon of larger muscle fiber bundle gaps caused by pre-slaughter stress is common in fish. The muscle fiber gap in muscle tissue of chub mackerel, Atlantic salmon, and silver carp under stress conditions was significantly increased compared with that of unstressed fish [12,23,44]. The myofibril density and collagen contents in muscle are positively related to the degree of flesh firmness and water-holding capacity, and a significant correlation relationship between flesh firmness and collagen contents in the flesh was reported [36,45,46]. Therefore, the breakage of myofibril and the degradation of intermuscular connective tissue synergically contributed to the flesh softening of shrimp after WFT, which were probably the major reasons other than protein oxidation that led to flesh softening and drip loss, eventually resulting in the deterioration of flesh quality [9].

## 5. Conclusions

In summary, cathepsin, calpain, and gelatinase in the muscle of shrimp were activated during WFT. However, no significant changes in the activity of caspase-3 and the muscle cell apoptosis rate were detected in shrimp muscle cells after WFT. As a result, the breakage of myofibril and the degradation of intermuscular connective tissue occurred under hydrolysis by cathepsins (cathepsin B and cathepsin L), calpains (μ-calpain and m-calpain), gelatinases (gelatinase-1, gelatinase-2, and gelatinase-3). Consequently, myofibril fragments and muscle fiber bundle gap enlargement were found in the muscle of shrimp after WFT. We indicated that WFT had a stronger activation effect on endogenous proteases in muscle than WT. The findings proposed some novel insights into the mechanisms of flesh quality decline in shrimp under transport stress other than protein and lipid oxidation, and glycolysis. These results provide new information for water-free transportation strategies and will aid in the development of commercial practices.

## Data Availability

The original contributions presented in the study are included in the article, and further inquiries can be directed to the corresponding authors.
